# Genetic Diversity, Population Structure and Linkage Disequilibrium in Elite Chinese Winter Wheat Investigated with SSR Markers

**DOI:** 10.1371/journal.pone.0044510

**Published:** 2012-09-05

**Authors:** Xiaojie Chen, Donghong Min, Tauqeer Ahmad Yasir, Yin-Gang Hu

**Affiliations:** 1 State Key Laboratory of Crop Stress Biology in Arid Areas, College of Agronomy, Northwest Agricultural and Forestry University, Yangling, Shaanxi, China; 2 Institute of Water Saving Agriculture in Arid Regions of China, Northwest Agricultural and Forestry University, Yangling, Shaanxi, China; Morehouse School of Medicine, United States of America

## Abstract

To ascertain genetic diversity, population structure and linkage disequilibrium (LD) among a representative collection of Chinese winter wheat cultivars and lines, 90 winter wheat accessions were analyzed with 269 SSR markers distributed throughout the wheat genome. A total of 1,358 alleles were detected, with 2 to 10 alleles per locus and a mean genetic richness of 5.05. The average genetic diversity index was 0.60, with values ranging from 0.05 to 0.86. Of the three genomes of wheat, ANOVA revealed that the B genome had the highest genetic diversity (0.63) and the D genome the lowest (0.56); significant differences were observed between these two genomes (P<0.01). The 90 Chinese winter wheat accessions could be divided into three subgroups based on STRUCTURE, UPGMA cluster and principal coordinate analyses. The population structure derived from STRUCTURE clustering was positively correlated to some extent with geographic eco-type. LD analysis revealed that there was a shorter LD decay distance in Chinese winter wheat compared with other wheat germplasm collections. The maximum LD decay distance, estimated by curvilinear regression, was 17.4 cM (r^2^>0.1), with a whole genome LD decay distance of approximately 2.2 cM (r^2^>0.1, P<0.001). Evidence from genetic diversity analyses suggest that wheat germplasm from other countries should be introduced into Chinese winter wheat and distant hybridization should be adopted to create new wheat germplasm with increased genetic diversity. The results of this study should provide valuable information for future association mapping using this Chinese winter wheat collection.

## Introduction

Wheat (*Triticum aestivum* L.) is one of the most important cereal crops worldwide. In China, wheat is grown on about 24 million hectares with a total annual production of 115 million tons and an average yield of 4.75 tons ha^-1^. Winter wheat occupies about 93% of the area planted in wheat and comprises approximately 94% of total wheat production in 2010 (http://faostat.fao.org/site/567/DesktopDefault.aspx?PageID=567#ancor). Genetic diversity is one of the most important factors for crop improvement. Over the past 60 years, wheat breeders have made remarkable progress in improving grain yield, disease resistance, quality and agronomic performance by using excellent germplasm resources. The recurrent use of a few elite germplasm lines as parental stock, however, has led to a decrease in genetic diversity and has narrowed the genetic base for wheat improvement [1]. The degree of genetic diversity found in contemporary germplasm from breeding programs may indirectly reflect the level of genetic progress achievable in future cultivars. Evaluation of wheat genetic diversity is therefore essential to the effective use of genetic resources in breeding programs.

Knowledge of genetic diversity is important for understanding the extent of genetic variability in existing plant material. Various types of markers can be used for genetic diversity estimation in wheat germplasm. In the past, morphological traits and physiological indexes were widely used for assessing genetic diversity, even though they are influenced by the environment and thus cannot be evaluated accurately. More recently, DNA molecular markers have been increasingly exploited for this purpose. They can be used for marker-assisted selection when tightly linked to target genes, and can also be employed to investigate levels of genetic diversity among categories such as cultivars and closely related species in germplasm banks [2], [3]. SSRs (Simple Sequence Repeats), which are among the most important molecular markers, are abundant, highly polymorphic, genome specific, codominant in nature, and show a fairly even distribution over the genome. SSRs have found application in analyses of genetic diversity and population structure, gene mapping, and assisted selection for crop improvement [4]–[7].

Linkage disequilibrium (LD), or nonrandom association of alleles between linked or unlinked loci, is becoming increasingly important for identifying genetic regions associated with agronomic traits [8]–[10]. Assessing relatedness among accessions is an important prerequisite for the identification of core germplasm collections suitable for optimizing association studies [11]. The presence of population structure has been widely documented in most studies investigating the diversity of elite crop germplasm, especially in self-pollinating cereals [7], [12]. In fact, the characterization of population structure within germplasm collections is critical to identify and correctly interpret the associations between functional and molecular diversity [13], [14].

Estimates of LD decay in wheat at the whole genome level and with large genetic representations of wheat genotypes are of great value. From an analysis of 242 genomic SSRs among 43 elite US wheat cultivars, it was reported that genome-wide LD estimates were generally less than 1 cM for genetically linked locus pairs, and that most of the LD was between loci less than 10 cM apart [15]. A collection of 189 bread wheat accessions genotyped at 370 loci and 93 durum wheat accessions genotyped at 245 loci were used to examine LD across the genome, and it was found that LD mapping of wheat could be performed with SSR markers to a resolution of less than 5 cM [16]. Estimates of LD at the chromosome level have also been reported. Researchers found consistent LD of less than 1 cM for chromosome 2D and about 5 cM in the 5A centromeric region using 33 and 20 SSR markers, respectively [17]. In another investigation [18], chromosome 3B had a lower diversity than the average for the entire B-genome. LD was weak in all studied accessions, and marker pairs with significant LD were generally concentrated around the centromere in both arms or at distal positions on the short arm.

In this study, a representative collection of 90 elite winter wheat accessions from the major wheat production regions of northern China were analyzed using 269 SSR loci distributed over all 21 chromosomes. Our objectives were to estimate the levels of genetic diversity and LD decay distance, and to characterize the population structure of this Chinese winter wheat germplasm collection. The results are intended to provide a molecular basis for understanding Chinese winter wheat genetic diversity, put forward suggestions for further improvement of wheat, and serve as a resource for association mapping using this collection.

## Results

### Diversity of SSR Loci in the Winter Wheat Collection

Using 269 SSR markers, genetic diversity of the Chinese winter wheat collection was characterized and evaluated at the genome level (Table 1, Table S3). Among the 269 SSR loci, a total of 1,358 alleles were detected, ranging from 2 to 10 per locus with a mean genetic richness of 5.05 (Table 1). Genetic diversity index values ranged from 0.05 to 0.86, and the mean genetic diversity index of 0.60 indicated low levels of polymorphism in this winter wheat collection. There were 375 (27.61%) rare alleles with a frequency of less than 5%, indicating that many new alleles were present in this Chinese winter wheat collection.

**Table 1:** Genetic diversity at genome level of the Chinese winter wheat revealed by 269 SSR markers.

Genome	No. of loci	No. of alleles	Mean allele number (range)	Genetic diversity (range)	No. of rare alleles (<5%)
A	90	499	5.54 (2–10) A	0.60 (0.05–0.84) AB	158
B	92	493	5.36 (2–10) A	0.63 (0.13–0.85) A	136
D	87	366	4.21 (2–9) B	0.56 (0.11–0.86) B	81
Whole genome	269	1358	5.05 (2–10)	0.60 (0.05–0.86)	375

The same letter within the fourth column indicates the difference is not significant as determined by the least significant difference (LSD) test at p<0.01.

The 269 SSR loci were evenly distributed between the A, B and D genomes of wheat (90, 92 and 87 alleles, respectively) (Table 1). Average genetic richness was 5.54 for the A genome, 5.34 for B, and 4.21 for D, and the genetic diversity index for the three genomes was 0.63 (B), 0.60 (A) and 0.56 (D). ANOVA revealed that the B genome had the highest genetic diversity and the D genome the lowest; significant differences were observed between these two genomes (P<0.01).

Comparison of genetic diversity among the seven homoeologous groups of wheat (Table 2) revealed that the average genetic richness for homoeologous groups 3 (5.4), 5 (5.25) and 7 (5.24) was higher than that for groups 2 (5.0), 4 (5.0), 1 (4.81) and 6 (4.48). In addition, the average genetic diversity index for homoeologous groups 2 (0.62), 5 (0.62), 6 (0.6) and 7 (0.6) was higher than that for 3 (0.58), 4 (0.58) and 1 (0.57).

**Table 2:** Genetic diversity in 7 homoeologous groups of the Chinese winter wheat collection as revealed by SSR markers.

Homoeologousgroup	No. of loci	No. of alleles	Mean allele numberMean (range)	Genetic diversity Mean (range)
1	42	202	4.81 (2–9)	0.57 (0.05–0.85)
2	46	230	5.00 (2–9)	0.62 (0.14–0.83)
3	43	232	5.40 (2–10)	0.58 (0.12–0.83)
4	26	130	5.00 (2–10)	0.58 (0.13–0.85)
5	44	231	5.25 (2–10)	0.62 (0.15–0.86)
6	31	139	4.48 (2–10)	0.60 (0.20–0.83)
7	37	194	5.24 (2–10)	0.60 (0.20–0.85)

The number of loci, number of alleles, average genetic richness and genetic diversity index for the 21 wheat chromosomes are shown in Table 3. For all chromosomes, there were 8–19 loci and 31–110 alleles; average genetic richness ranged from 3.44 to 6.29 and diversity index values varied from 0.51 to 0.66. The highest genetic richness (6.29) as well as the highest genetic diversity index (0.66) was observed in chromosome 3B. The two indices for chromosome 6D were the lowest (average genetic richness  = 3.44; genetic diversity index  = 0.51). The three highest genetic richness values were observed in 3B (6.29), 4A (5.9) and 7A (5.89); and the three lowest were observed in 6D (3.44), 4D (3.88) and 7D (4.08). The three highest genetic diversity index values were observed in chromosomes 3B (0.66), 5B (0.65) and 6B (0.65), and the four lowest were observed in chromosomes 6D (0.51), 3D (0.53), 4D (0.53) and 7D (0.53).

**Table 3:** Genetic diversity of each chromosome in the Chinese winter wheat collection as revealed by SSR markers.

Chr.	No. of marker	No. of alleles	Mean allele number (range)	Genetic diversity (range)
1A	15	83	5.53 (2–9)	0.54 (0.05–0.84)
1B	12	51	4.25 (2–6)	0.60 (0.31–0.78)
1D	15	68	4.53 (2–7)	0.56 (0.11–0.85)
2A	13	73	5.62 (2–9)	0.64 (0.31–0.83)
2B	18	92	5.11 (2–7)	0.62 (0.30–0.83)
2D	15	65	4.33 (2–7)	0.61 (0.14–0.83)
3A	14	68	4.86 (2–8)	0.53 (0.13–0.80)
3B	16	105	6.56 (3–10)	0.67 (0.39–0.82)
3D	13	59	4.54 (2–9)	0.53 (0.12–0.83)
4A	10	59	5.90 (4–10)	0.62 (0.13–0.80)
4B	8	40	5.00 (3–8)	0.57 (0.13–0.85)
4D	8	31	3.88 (2–6)	0.53 (0.35–0.73)
5A	19	110	5.79 (3–10)	0.63 (0.15–0.84)
5B	10	58	5.80 (3–8)	0.65 (0.32–0.81)
5D	15	63	4.20 (2–8)	0.59 (0.18–0.86)
6A	10	53	5.39 (2–10)	0.63 (0.20–0.83)
6B	12	55	4.58 (3–6)	0.65 (0.35–0.75)
6D	9	31	3.44 (2–6)	0.51 (0.21–0.73)
7A	9	53	5.89 (2–9)	0.64 (0.32–0.83)
7B	16	92	5.75 (2–10)	0.64 (0.20–0.85)
7D	12	49	4.08 (2–7)	0.53 (0.24–0.80)

**Figure 1:**
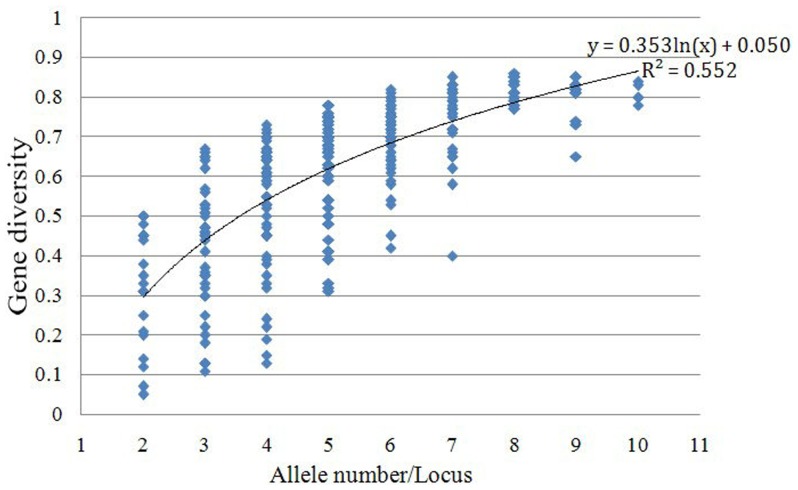
Scatter plot of gene diversity vs. number of alleles per locus.

The genetic diversity index was usually positively correlated with number of alleles and there was a positive linear relationship between the two indices within a given range. Simple correlation analysis (Fig. 1) indicated that the genetic diversity index was significantly and positively correlated with the number of alleles (r  = 0.72, P<0.001). Genetic diversity (y) could be estimated by a curvilinear regression equation with independent variables of allele number per locus (x), i.e., y  = 0.353 ln(x) +0.050 (1< x <11), R^2^ = 0.552.

### Population Structure Analysis

**Figure 2:**
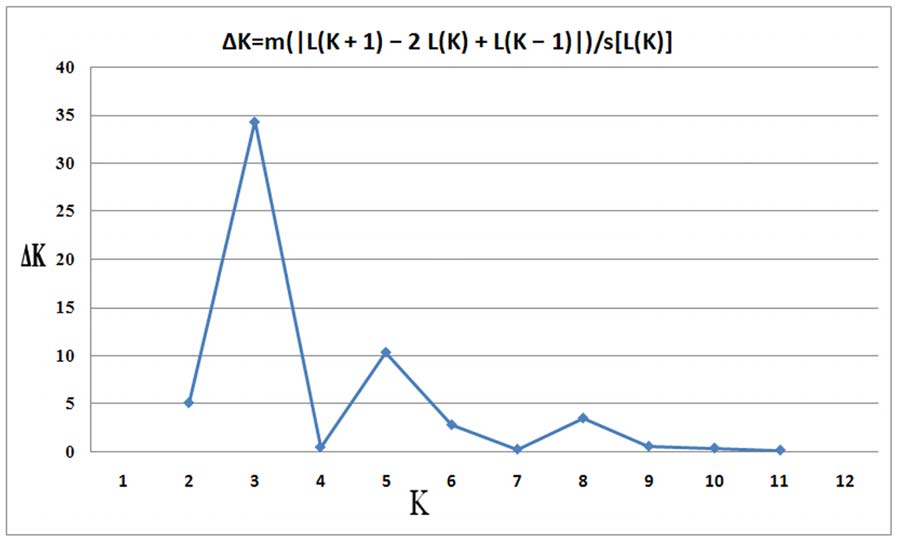
STRUCTURE estimation of the number of populations for K ranging from 1 to 12 by delta K values ( Δ**K).**

**Figure 3:**
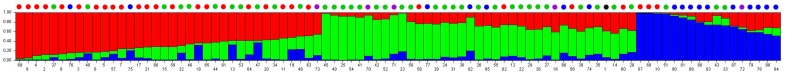
Three subgroups inferred from STRUCTURE analysis. The vertical coordinate of each subgroup indicates the membership coefficients for each individual, and the digits on the horizontal coordinate represent the accessions corresponding to Table S1. Red zone: SG 1; Green zone: SG 2; Blue zone: SG 3. The colored spots represent the geographic eco-type information of accessions. Red spot: cultivars from northern winter wheat region; Green spot: cultivars from Huang-huai winter wheat region; Blue spot: landraces and introduced germplasm; Purple spot: cultivars from Southwestern winter wheat region; Black spot: cultivars from Yangtze River winter wheat region.

Population structure of the 90 accessions was estimated using STRUCTURE V2.3.3 software based on 269 whole-genome unlinked SSR markers. The number of subpopulations (K) was identified based on maximum likelihood and delta K (ΔK) values, with accessions falling into three subgroups (Fig. 2). Using a membership probability threshold of 0.60, 24 accessions were assigned to subgroup (SG) 1, 27 accessions to SG 2, 13 accessions to SG 3 and 26 accessions were retained in the admixed group (AD). With the maximum membership probability, 36 accessions were assigned to SG 1, 37 accessions to SG 2 and 17 accessions to SG 3 (Fig. 3). The relationship between subgroups derived from STRUCTURE clustering and geographic eco-types of modern cultivars from Northern and Huang-huai winter wheat regions was further analyzed. SG 1 comprised 22 cultivars from the Northern and 11 from the Huang-huai winter wheat region; SG 2 consisted of 4 Northern and 24 Huang-huai cultivars; and SG 3 included 3 from each region. Among these 67 cultivars, 46 (68.66%) could be differentiated using STRUCTURE (Fig. 3). This indicates that, to some extent, the population structure assigned by STRUCTURE clustering was positively correlated with geographic eco-types of the accessions.

**Figure 4:**
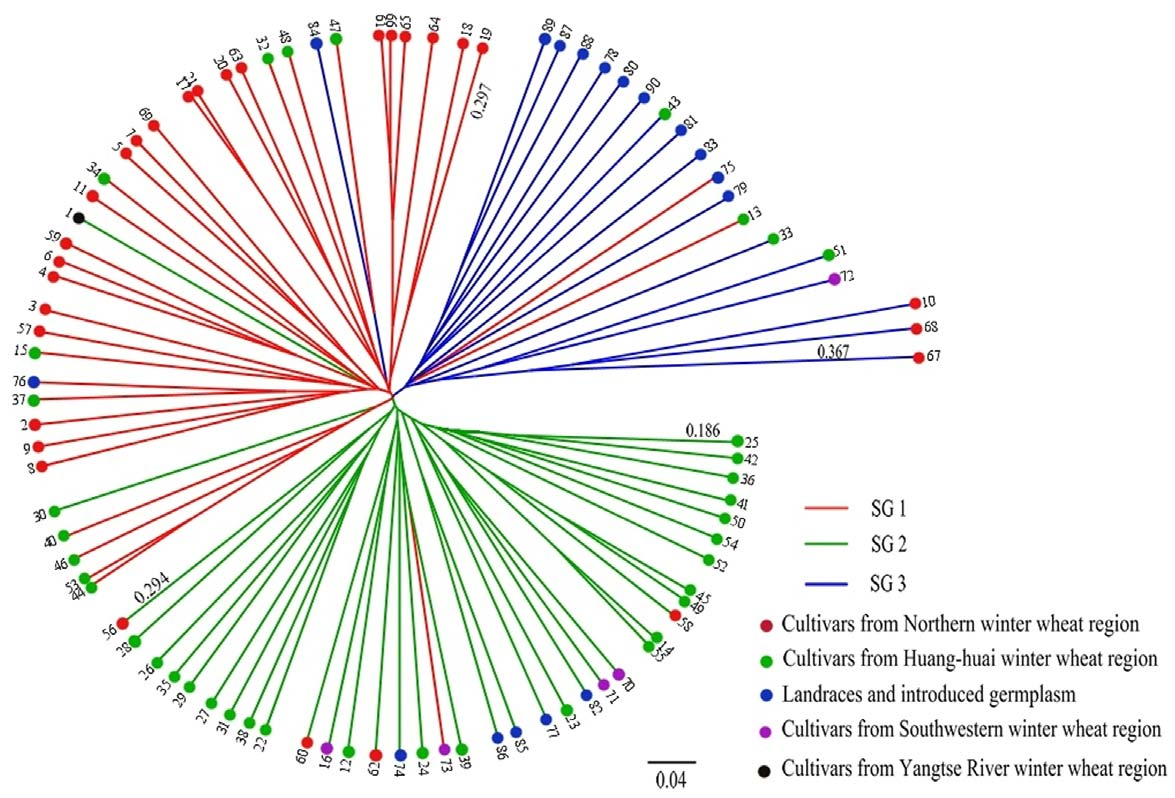
Dendrogram of 90 Chinese winter wheat accessions by UPGMA cluster analysis. SG 1, SG 2 and SG 3 are the three subgroups identified by STRUCTURE assigned with the maximum membership probability. The different colored lines represent the three subgroups inferred by STRUCTURE analysis. The different colored spots represent the geographic eco-types of accessions.

UPGMA cluster analysis also clearly divided the 90 accessions into three groups when the genetic distance was about 0.294. Subpopulations 1–3 comprised 36, 36 and 18 accessions, respectively (Fig. 4). The dendrogram of the 90 accessions also revealed that subpopulation 1 was genetically more similar to subpopulation 2 than to subpopulation 3. Subpopulations 1 and 2 clustered into one class when the genetic distance was approximately 0.297, and all 90 accessions were grouped into a single cluster at a genetic distance of about 0.367. Compared to the subgroups derived from STRUCTURE, the assignments of 84 accessions (93.33% of the total) by UPGMA clustering were consistent with their assignments using STRUCTURE.

**Figure 5:**
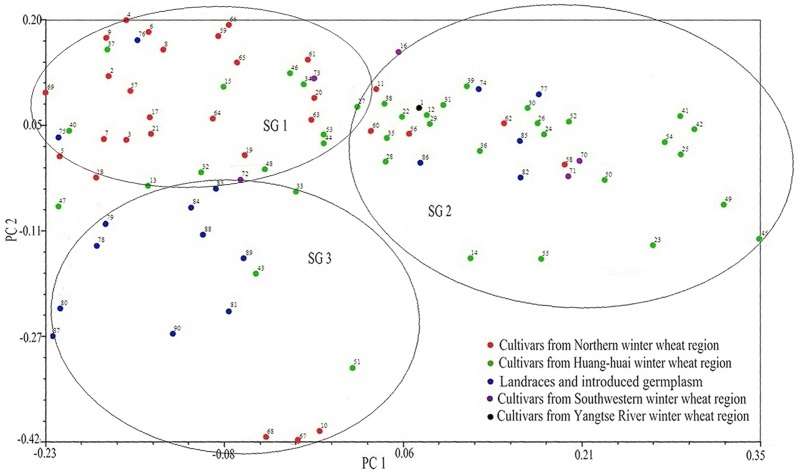
Principal coordinate analysis of 90 Chinese wheat winter accessions based on 269 SSR markers. SG 1, SG 2 and SG 3 are the three subgroups identified by STRUCTURE assigned with the maximum membership probability. The different colored spots represent the geographic eco-types of accessions.

Principal coordinate analysis separated the 90 accessions into three major groups, which was consistent with assignments generated by STRUCTURE and UPGMA clustering (Fig. 5). The accessions belonging to SG 1 (as inferred by STRUCTURE analysis) were mainly distributed in the upper left portion of the resulting plot, with SG 2 distributed in the upper right and SG 3 in the lower left. The distribution of accessions of SG 1 and SG 2 were more tightly clustered than SG 3, indicating that accessions in SG 3 had higher diversity than SG 1 and SG 3. Landraces and introduced germplasm were more widely scattered than modern cultivars, indicating that the landraces and introduced germplasm had higher diversity.

### Linkage Disequilibrium Estimation in Chinese Winter Wheat

The SSR data (90 loci from the A genome, 92 from the B genome, and 87 from the D genome) were used to evaluate the extent of LD on a whole genome level (Table 4). Across all 296 loci, 36,046 locus pairs were detected in the Chinese winter wheat collection. Among them, 1,340 locus pairs (3.72%) were revealed at the P<0.001 level, and 79 locus pairs (0.22%) were found at r^2^>0.1 and P<0.001. Among all locus pairs, 1,692 linked locus pairs were detected. Among linked locus pairs, 162 (9.57%) were in LD at the P<0.001 level (Table S4). Of all significant pairs (P<0.001), 21 (1.24% of 1,692) showed an r^2^ value greater than 0.1. Higher LD was observed in linked locus pairs than in unlinked locus pairs. Only 58 unlinked locus pairs (0.17% of 34,354) showed an r^2^ value greater than 0.1.

**Table 4:** Linkage disequilibrium (LD) patterns of SSR locus pairs in the Chinese winter wheat collection.

	Linked locus pairs				Unlinked locus pairs	Total locus pairs
Genome	A	B	D	Whole gnome		
Observed	571	598	523	1692	34354	36046
P<0.00,1%	64 (11.21)	82 (13.71)	16 (3.43)	162 (9.57)	1178 (3.43)	1340 (3.72)
r^2^>0.1,%	7 (1.23)	8 (1.34)	7 (1.34)	22 (1.30)	74 (0.22)	96 (0.27)
P<0.001, r^2^>0.1,%	7 (1.23)	8 (1.34)	6 (1.15)	21 (1.24)	58 (0.17)	79 (0.22)

**Figure 6:**
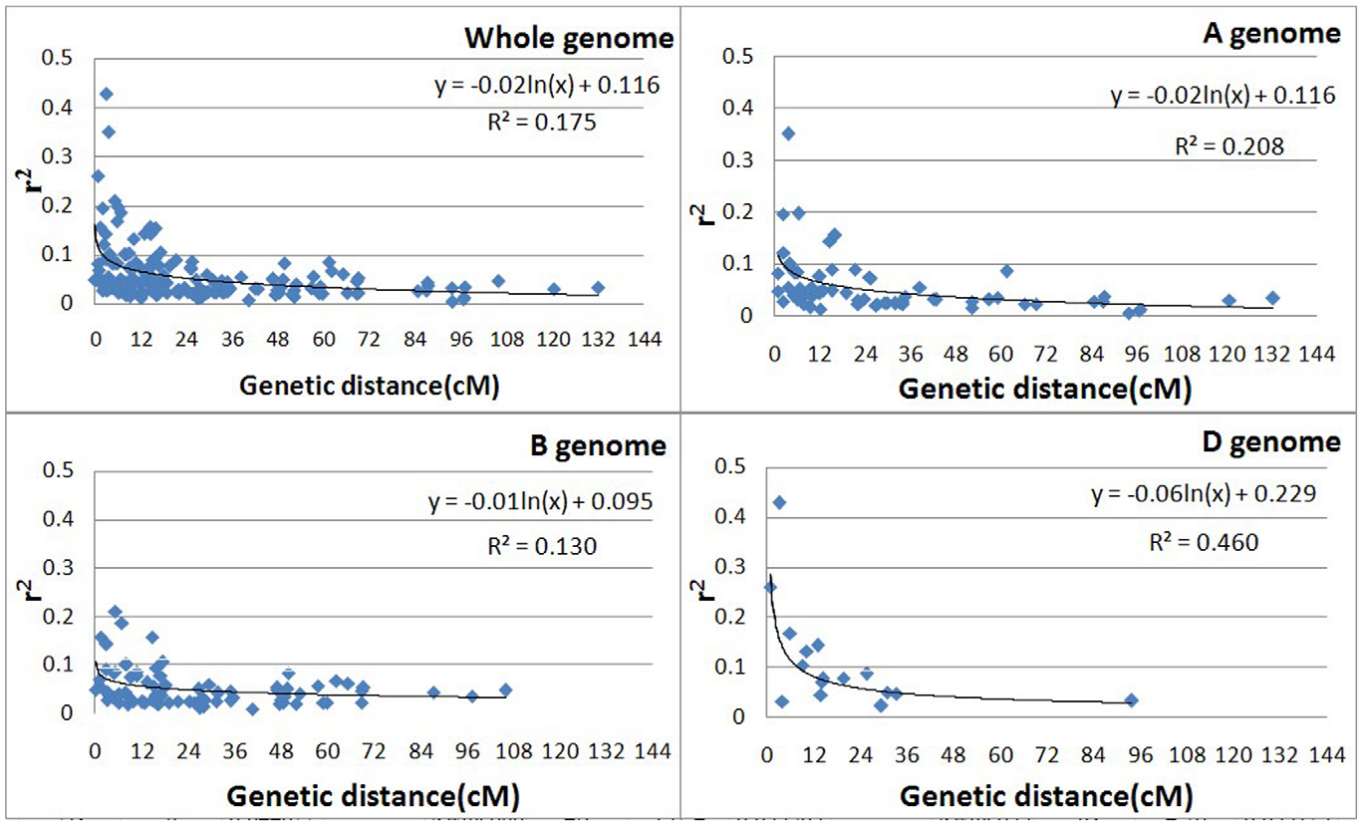
Scatter plot of significant r^2^ values and genetic distance (cM) (P<0.001) of locus pairs on A, B, D and whole genomes in Chinese winter wheat.

To reveal LD decay distances in the Chinese winter wheat collection on a whole genome scale, we generated LD decay scatter plots of syntenic r^2^ vs. genetic distance (cM) and estimated LD decay distances using curvilinear regression (Fig. 6). As the inter-locus distance increases, the r^2^ value decreases. The LD (r^2^>0.1) decay distance extended maximally to 17.4 cM. An LD decay distance of about 2.2 cM was estimated for the whole genome, and LD decay distances for A, B, and D genomes were approximately 2.2, 0.6, and 8.6 cM, respectively (Fig. 6).

## Discussion

### Genetic Diversity in Chinese Winter Wheat

SSR markers have been extensively used to detect variability in wheat genotypes and to evaluate their genetic diversity. Compared with previous reports, the genetic diversity of the 90 Chinese winter wheat accessions in this study was at a lower level, as reflected by the mean genetic diversity value (0.6) and average number of alleles per locus (5.05). Other researchers have reported averages of 4.81 to 18.1 alleles per locus and mean genetic diversity values ranging from 0.46 to 0.77 in various wheat collections [5], [6], [7], [17], [19], [20], [21]. A study of 205 U.S. hard and soft wheat accessions uncovered a genetic diversity value of 0.54 and an average of 7.2 alleles per locus [6]. The genetic diversity of 559 French wheat accessions was evaluated using 42 SSR markers; 14.5 alleles were identified per locus and a polymorphic information content (PIC) value of 0.66 was obtained [21]. A worldwide collection of 998 accessions was studied, revealing a gene diversity value of 0.77 and 18.1 alleles per locus at 26 SSR loci [5]. The genetic diversity of 250 Chinese bread wheat accessions, including 93 modern varieties and 157 landraces, was also investigated [7]. The authors reported an average of 13.1 alleles per locus and a mean genetic diversity of 0.65, and found that landraces had a higher genetic diversity (0.64) than modern varieties (0.628). In our study, genetic diversity was lower because only 11 landraces were included among the 90 accessions. Most genetic diversity analyses on wheat have been undertaken at the whole genome level. In this study, significant difference in genetic diversity among the three genomes was observed. The B genome showed the highest diversity (0.63) and the D genome the lowest (0.56). Similarly result was reported, the average genetic richness, from highest to lowest, was B>A>D, and genetic diversity indexes were ordered B>D>A [22]. Therefore, we have thus determined that there is a lower level genetic diversity in Chinese modern wheat varieties, especially for the D and A genomes. Consequently, introduction of wheat germplasm from other countries into Chinese winter wheat breeding programs has the potential to enhance the genetic diversity of breeding materials. Furthermore, the augmentation of wheat germplasm with chromosomal fragments from wheat-related species via distant hybridization and embryo rescue would greatly increase the genetic diversity of wheat. The utilization of 1B/1R translocation lines was seen as one of the most successful cases in wheat breeding. The genetic diversity of a worldwide set of 161 winter and spring triticale lines was investigated by genotyping with high-density DArT markers, and higher polymorphic information content (PIC) in triticale was observed while compared to wheat [23]. As triticale is closely related to wheat with the A and B genome, it may be more advantageous than other wheat-related species for wheat germplasm innovation.

### Population Structure

Population structure is one of several important factors that strongly influence LD. The presence of population stratification and an unequal distribution of alleles within groups can result in nonfunctional spurious associations [8]. Population structure analyses have indicated that wheat accessions can be categorized by geographical origin or be divided into landraces and modern varieties. Using the maximum membership probability in STRUCTURE, 36 accessions were assigned to SG 1, 37 to SG 2, and 17 to SG 3 (Fig. 3). UPGMA cluster analysis demonstrated that the 90 accessions could also be clearly divided into three groups, and the assignment of 84 accessions (93.33% of the total) by UPGMA clustering was consistent with their classification using STRUCTURE (Fig. 4). Principal coordinate analysis also separated the 90 accessions into three major groups (Fig. 5). These results confirmed that population structure, comprising three subgroups, actually existed within the 90 accessions. In this study, 46 of the 67 cultivars from Northern and Huang-huai winter wheat regions (68.66%) could be assigned to two subgroups derived from STRUCTURE clustering; similar results were obtained by UPGMA cluster and principal coordinate analyses. This indicates that the population structure was positively correlated with geographic eco-type.

With a membership probability threshold of 0.60, 24 accessions were assigned by STRUCTURE to SG 1, 27 to SG 2, 13 to SG 3, and 26 to the admixed group (AD) (Fig. 3). According to UPGMA cluster analysis results, all 90 accessions grouped together into one class when the genetic distance was about 0.376 (Fig. 4). This suggests a low level of genetic diversity, consistent with the genetic diversity estimation obtained using PowerMarker software.

Although there were only 11 landraces and 6 introduced germplasm representatives in this study, principal coordinate analysis indicated that they differed to some extent from cultivars (Figs. 3, 4 and 5). In addition, UPGMA cluster and principal coordinate analyses revealed that the sampled landraces and introduced germplasm varieties had higher diversity than modern cultivars.

Population structure is an important component in association mapping analyses between molecular markers and traits because it may reduce both type I and II errors. All three population structure approaches, i.e., STRUCTURE, UPGMA, and principal coordinate analysis, showed a more-or-less positive correlation between population structure and geographic eco-types. Because the population structure revealed by STRUCTURE analysis based on many SSR markers distributed throughout the wheat genome was more accurate than using geographic eco-type information, this program was concluded to be the most suitable for investigating population genetic structure.

### Linkage Disequilibrium

It is important to estimate LD decay distances when carrying out association mapping. The LD decay distance determines the marker density needed to effectively associate genotypes with traits and influences the precision of association mapping. A higher level of LD might be expected in hexaploid wheat than in maize and sorghum because of the rapid rate of inbreeding in wheat with a high degree of self-pollination [8].

Numerous studies in wheat have reported LD decay distances. Mean genome-wide LD decay was estimated to be 10 cM (r^2^>0.1) in 205 U.S. elite wheat breeding lines [6]. In another study [7], Chinese modern varieties displayed a wider average LD decay than landraces across the whole genome for locus pairs with r^2^>0.05 (P<0.001); mean LD decay distance for 157 landraces at the whole genome level was 5 cM compared with 5–10 cM for the 93 modern varieties investigated. For 189 bread wheat and 93 durum wheat accessions, the LD between adjacent locus pairs extended (r^2^>0.2) approximately 2–3 cM on average, and it was suggested that LD mapping of wheat could be performed with SSRs to a resolution of <5 cM [16]. From an analysis of 242 genomic SSRs among 43 elite U.S. wheat cultivars, it was reported that genome-wide LD estimates generally were less than 1 cM for genetically linked locus pairs with r^2^>0.2 (P<0.01), and that most LD was less than 10 cM apart between loci [15]. Recently, LD decay distances of 161 diverse winter and spring triticale genotypes assayed was estimated using 2,079 genome-wide distributed DArT markers; a similar LD decay distance within approximately 12 cM was observed for the A and B genome and a lower LD with approximately 5 cM was found in the R genome [23]. In our study, the maximum LD decay distance (r^2^>0.1) was 17.4 cM, with an average LD decay distance of about 2.2 cM for the whole Chinese winter wheat genome (Fig. 6). This overall LD decay distance was shorter than that reported for other studies. As may been seen from the above-mentioned studies, reported LD decay distances vary greatly from one study to the next. These differences are due both to variations in material type and quantity as well as differences in r^2^ values (i.e., r^2^>0.05, 0.1, or 0.2) used for the estimations. Most LD decay distances were estimated with r^2^ = 0.1.

Our results also demonstrate that LD decay distances differ among the three wheat genomes – the LD decay distance in A, B and D genomes of wheat was about 2.2, 0.6 and 8.6 cM, respectively (Fig. 5). The highest extent of LD was observed in the wheat D genome than A and B genomes. Similar result was reported, the linkage disequilibrium (LD) evaluated on 1536 SNPs among 478 spring and winter wheat (*Triticum aestivum*) cultivars from 17 populations across the United States and Mexico revealed that the highest extent of significant LD was observed in the wheat D genome, followed by lower LD in the A and B genomes [24]. Major concerns with LD analyses were at the chromosome level as reported [7], [17], [18].

In summary, this study demonstrates that genetic diversity in Chinese winter wheat, especially on D and A genomes, is lower than that of many other wheat germplasm collections. Continuous introduction of wheat germplasm from other countries and the adoption of distant hybridization technology to create new wheat germplasm are necessary to increase genetic diversity. The population structure revealed using STRUCTURE analysis of many SSR markers distributed throughout the wheat genome was more accurate than using geographic eco-type information and was available for investigating population genetic structure. LD analysis revealed that LD decay distance is shorter in Chinese winter wheat compared with other wheat collections. The development of high-density markers in these varieties will thus be required for association mapping.

## Materials and Methods

### Plant Materials

To ensure a broadly representative sampling of Chinese winter wheat, 328 Chinese winter wheat accessions were grown during the 2009–2010 growing season on the experimental farm of the Institute of Water Saving Agriculture in Arid Areas of China, Northwest A & F University, Yangling, Shaanxi, China. A representative collection of 90 cultivars and lines was chosen by discarding accessions with similar pedigrees as well as those that failed to mature normally. The name, pedigree (if available), origin and geographic eco-type of these 90 accessions are listed in Table S1. The accessions comprised 73 cultivars and 11 landraces from 13 provinces of major wheat production regions in China, as well as 6 varieties introduced from other countries. Of the 73 modern cultivars, there were 38 from the Huang-huai winter wheat region, 29 from the Northern region, 5 from the Southwestern region, and 1 from the Yangtze River region. The 11 landraces, which have been widely used in breeding programs, were obtained from Henan, Shaanxi and Sichuan provinces. The six introduced cultivars were Jubilejna 1, Early Premium, Lovrin 10, Akagomughi, Norin 10 and Suwon 86, all important parents used in Chinese wheat breeding.

### SSR Markers and Genotyping

Genomic DNA was extracted from approximately 200 mg fresh leaf tissue using the cetyltrimethylammonium bromide (CTAB) method [25]. A total of 269 SSR markers, which were distributed evenly over the 21 wheat chromosomes, were selected and synthesized according to the information available in the GrainGenes database (http://wheat.pw.usda.gov/GG2) (Table S2).

PCR amplifications were carried out in 20 µL reaction volumes containing 2 µL template DNA, 2 µL 10X buffer, 0.1 µL of 5 units µL^−1^ Taq DNA polymerase, 0.4 µL 10 mM dNTPs, and 2 µL each of 2.5 µM primer. PCR protocols consisted of 35 cycles of 94°C for 45 s, an annealing temperature appropriate for the particular primer pair for 45 s, and 72°C for 60 s, and a final extension step of 72°C for 10 min. PCR products were analyzed by 8% polyacrylamide gel electrophoresis (PAGE) and visualized by silver staining.

### Genetic Diversity Estimation

For each SSR locus, polymorphic bands were scored as 1 or 0 for presence or absence of the bands at the same mobility, respectively. Gene diversity, or PIC [26], was calculated for each marker using Nei’s simplified formula [27]:
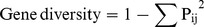
where P_ij_ is the frequency of the j-th allele for the i-th locus summed across all alleles for that locus. The program PowerMarker v3.25 was used to calculate allele number, allele frequency, and gene diversity of each locus [28].

### Population Structure Analysis

The population structure of the 90 winter wheat accessions was assessed using the model-based (Bayesian clustering) method implemented in STRUCTURE v2.3.3 (http://pritch.bsd.uchic-ago.edu/structure.html) [29]. The number of subgroups (K) was set from 1 to 12 based on models characterized by admixture and correlated allele frequencies. For each K, ten runs were performed separately, with 100,000 iterations carried out for each run after a burn-in period of 10,000 iterations. A K value was selected when the estimate of lnPr(X|K) peaked in the range of 1 to 12 subpopulations. The model choice criterion to detect the most probable value of K was ΔK, which is an ad hoc quantity related to the second order change of the log probability of data with respect to the number of clusters inferred by STRUCTURE [30]. The run with the maximum likelihood was applied to subdivide the varieties into different subgroups using a membership probability threshold of 0.60 as well as the maximum membership probability among subgroups. Those varieties with less than 0.60 membership probabilities were retained in the admixed group (AD). Results from STRUCTURE were displayed using Distruct 1.1 [31].

To examine genetic relationships, UPGMA cluster analysis was carried out with PowerMarker using the genetic similarity matrix, and the resulting dendrogram was drawn by FigTree v1.3.1 [26], [32]. Using NTSYS-pc v2.1 [33], principal coordinate analysis of the variance-covariance matrix calculated from marker data was also employed to reveal relationships among the 90 accessions.

### Linkage Disequilibrium Estimation

Linkage disequilibrium estimates and significance for each pair of SSR loci were evaluated using TASSEL v2.1 (www.maizegenetics.net/) with 1000 permutations. The LD parameter r^2^ among loci, which is the squared correlation coefficient between two loci and which summarizes both mutational and recombination history, was calculated separately for unlinked loci on different chromosomes and for linked loci on the same chromosome (unlinked r^2^ and syntenic r^2^, respectively). Loci were considered to be in significant LD if P<0.001. LD decay scatter plots of syntenic r^2^ vs. genetic distance (cM) between markers were generated using SPSS18.0. LD decay was calculated according to the method described in [17].

## Supporting Information

Table S1 **Detailed information for the 90 accessions used in this study.**
(XLSX)

Table S2 **List of 269 SSR loci used in this study.**
(XLSX)

Table S3 **Summary statistics of the 269 SSR loci in this study.**
(XLSX)

Table S4 **Linkage disequilibrium (LD) of 162 linked locus pairs at the P<0.001 level.**
(XLSX)
